# Women's constructions of the 'right time' to consider decisions about risk-reducing mastectomy and risk-reducing oophorectomy

**DOI:** 10.1186/1472-6874-10-24

**Published:** 2010-08-05

**Authors:** A Fuchsia Howard, Joan L Bottorff, Lynda G Balneaves, Charmaine Kim-Sing

**Affiliations:** 1 School of Population and Public Health, University of British Columbia, Canada; 2 Faculty of Health and Social Development, University of British Columbia Okanagan, Canada; 3 School of Nursing, University of British Columbia, Canada; 4 British Columbia Cancer Agency & Faculty of Surgery, University of British Columbia, Canada

## Abstract

**Background:**

Women who are notified they carry a *BRCA1/2 *mutation are presented with surgical options to reduce their risk of breast and ovarian cancer, including risk-reducing mastectomy (RRM) and risk-reducing oophorectomy (RRO). Growing evidence suggests that a sub-group of women do not make decisions about RRM and RRO immediately following genetic testing, but rather, consider these decisions years later. Women's perspectives on the timing of these decisions are not well understood. Accordingly, the purpose of this research was to describe how women construct the 'right time' to consider decisions about RRM and RRO.

**Methods:**

In-depth interviews were conducted with 22 *BRCA1/2 *carrier women and analyzed using qualitative, constant comparative methods.

**Results:**

The time that lapsed between receipt of genetic test results and receipt of RRM or RRO ranged from three months to nine years. The findings highlighted the importance of considering decisions about RRM and RRO one at a time. The women constructed the 'right time' to consider these decisions to be when: (1) decisions fit into their lives, (2) they had enough time to think about decisions, (3) they were ready emotionally to deal with the decisions and the consequences, (4) all the issues and conflicts were sorted out, (5) there were better options available, and (6) the health care system was ready for them.

**Conclusions:**

These findings offer novel insights relevant to health care professionals who provide decision support to women considering RRM and RRO.

## Background

Genetic testing for mutations in the *BRCA1 *and *BRCA2 *genes has increasingly become available to individuals since the discovery of these genes over 14 years ago [1,2]. Those women found to carry *BRCA1/2 *mutations are at markedly increased probability of developing hereditary breast and ovarian cancer (HBOC), with their lifetime risk of breast cancer between 45% and 88%, and their risk of ovarian cancer ranging from 11% to 65% [3-5]. When unaffected women are notified that they have a *BRCA1/2 *mutation, they are presented with a range of HBOC risk-reducing options including risk-reducing mastectomy (RRM) and risk-reducing oophorectomy (RRO). However, there is growing evidence that a sub-group of women do not make decisions about RRM or RRO immediately after genetic testing, but rather, prolong these decisions for months or years [6-9]. Women's experiences with RRM and RRO have begun to be described [10-12], but their perspectives on the timing of these decisions are not well understood. The purpose of this study was to describe how women who carry *BRCA1/2 *mutations construct the 'right time' to consider decisions about risk-reducing surgeries.

## Background Literature

Women found to carry a *BRCA1/2 *mutation are faced with difficult decisions about how to manage their elevated HBOC risk. Breast cancer screening is recommended to these women as a means of identifying cancers at an early stage when the prognosis of treatment is good, thus reducing the risk of dying from cancer. The most effective means of preventing breast cancer is through RRM, the surgical removal of healthy breast tissue prior to the development of cancer, with the option of reconstructive surgery. However, RRM is considered controversial because of the potential for psychological harm. It is, thus, generally framed as a woman's personal decision to be discussed with her health care professional [13]. As there is no evidence that ovarian cancer screening is effective in reducing mortality, it is currently assumed that RRO is the best form of risk management. As such, RRO, the surgical removal of the fallopian tubes and ovaries, is commonly recommended to women once childbearing is complete [13].

Women are encouraged to make decisions about RRM and RRO when they receive their genetic test results. However, there is evidence that some women are delaying these decisions. In previous research, there were women who underwent RRO and/or RRM years after receiving their genetic test results [6-9]. In the largest of these studies, which included 297 women who had RRO and 113 women who had RRM, the average time from genetic testing to risk-reducing surgery was 1 year [9]. Yet, some of these women had RRO up to 7 years later and RRM more than 8 years later. For women in another study who previously had a mastectomy to treat breast cancer, the mean time that elapsed between their primary surgery and contralateral RRM was 3.5 years [14]. The amount of time between receipt of genetic test results and risk-reducing surgery has raised questions about why women are not acting promptly to reduce their risks, and concerns that the full benefits of genetic testing are not being realized.

It has been suggested that medical, physical, psychological, and social context factors influence the timing of decisions and use of risk-reducing options. Women's childbearing and menopausal status have been reported to have a substantial bearing on how women frame the optimal timing of these decisions, as have women's relationships and their responsibilities to family members, friends, and employers [7,15-18].

The psychological consequences of genetic testing, including anxiety, distress, and worry, may dramatically influence women's abilities to make risk-reducing decisions, as well as the nature of the decision-making process. Although these psychological symptoms decline over time for the majority of mutation carriers [19-22], significant psychological distress can persist for others [22,23]. Moreover, researchers have reported a positive relationship between distress, anxiety, and worry, and decisions about RR strategies [24-30].

When positive *BRCA1/2 *test results are disclosed, women are also faced with interpreting complex and emotion-laden information. Women have reported spending much time reviewing information and seeking additional sources of information and advice in an attempt to resolve their questions about the potential impact of RRM on their lives [31]. In one study, the lack of absolutes about when, or if, the women would develop HBOC led to uncertainty about the right time to make RRM and RRO decisions [32]. Moreover, *BRCA1/2 *carriers have expressed a need to consider relevant information in the context of their individual experiences, perceptions, and psychosocial needs [33].

It is also important to acknowledge that once women receive their genetic test results they may encounter barriers and delays accessing health services that are central to decisions about risk-reducing surgery and receipt of risk management choices. For example, in Canada obtaining consultations with specialized health care professionals may take months and even years, while in the United States the financial costs of health services or the lack of health insurance may be prohibitive. Women in rural settings may experience additional barriers to treatment including costs of travel and time away from employment [32].

Although previous research provides important insights related to risk management decision making, women's perspectives related to the timing of decisions has not been systematically studied. With few exceptions, the focus of research to date has been guided by theoretical perspectives and assumptions that have not adequately captured the complexity of women's decision making [18], or their perspectives on the importance of making timely decisions and factors that influence timing. A few studies have described women's decision-making processes about risk-reducing surgery as complex, dynamic, and prolonged [16,31]. In a case study that involved in-depth interviews with three women over one year, researchers tracked how women's thoughts and decisions about RRM changed over time, and the time they needed to achieve a level of comfort with decisions that they were able to follow through with [31]. It is essential that health care professionals consider women's perspectives on the 'right time' to make these decisions given the potential psychological implications of making untimely decisions, as well as the implications of delaying RRM and RRO with regard to women's morbidity and mortality. A better understanding of women's perspectives is also needed to guide the provision of RRM and RRO decision support, as well as the development of decision support interventions that address the complex needs faced by women at high risk for HBOC.

## Methods

This study was conducted in the context of a larger grounded theory study aimed at developing an in-depth theory of women's decision making regarding HBOC risk-reducing strategies [32]. Qualitative methodological approaches described by Charmaz [34], Strauss and Corbin [35], and Sandelowski [36] were used to develop descriptions of how women construct the 'right time' to consider decisions about RRM and RRO.

### Study Participants

The University of British Columbia behavioral research ethics board approved this research. Participants were recruited through a hereditary cancer program in British Columbia, Canada. Staff at the hereditary cancer program distributed a letter of invitation to potential study participants by mail or in-person at genetic counseling appointments following receipt of their genetic test results or the high-risk clinic. The women who expressed an interest in the study by mailing a signed consent to contact form to the research team were then contacted by telephone, wherein they were given further details about the research, screened for eligibility, and scheduled for an interview if they consented to participate. Women who received positive *BRCA1/2 *genetic test results, were older than 18 years of age, and English speaking, were included in this study. Women who were currently undergoing diagnostic testing for cancer or were receiving cancer treatments were excluded.

In this Canadian hereditary cancer program, women undergo genetic counseling before and after genetic testing, wherein they receive oral and written information about HBOC risk-reducing strategies. RRO is recommended to women once childbearing is complete, and RRM is not recommended, but rather, is framed as a personal decision to be discussed with a health professional. Women found to carry a *BRCA1/2 *genetic mutation are also advised to undergo breast cancer screening at the high-risk clinic every six months, and although formal decision support is not systematically provided during these appointments, risk-reducing surgery is often discussed. If women express an interest in risk-reducing surgery they are then referred to a surgeon for a consultation.

The demographic characteristics of the 22 participants are illustrated in Table 1. The mean age of participants was 51 years of age (ranging from 28 to 80 years). The majority of women were Caucasian, college or university educated, and employed either full- or part-time. Most of the women were married, and just over half had children. Five women had a history of breast cancer.

**Table 1:** Characteristics of Study Sample

Characteristics	Frequency (%)
Marital Status	
Married/common-law	17 (77)
Single/separated/divorced/widowed	5 (23)
Ethnicity	
Anglo-Saxon	11 (50)
Ashkenazi Jewish	6 (27)
Other	5 (23)
Education	
Some college and above	15 (68)
High school and below	7 (32)
Employment	
Employed	18 (82)
Unemployed/homemaker/retired	4 (18)
Household annual income (Canadian)	
Less than 40,000	4 (18)
$41,000 - 80,000	8 (36)
Greater than $80,000	10 (46)
Children	
Have children	12 (55)
No children	10 (45)
Time since genetic testing	
Less than 1 year	5 (23)
1-4 years	4 (18)
Greater than 5 years	13 (59)
Cancer history	
Previous breast cancer history	5 (23)
No previous breast cancer history	17 (77)

*Note*. N = 22

### Data Collection Procedures

In-depth interviews, lasting 45 to 90 minutes, were conducted at a time and place convenient to the participants following receipt of written informed consent. All interviews were digitally recorded and transcribed verbatim. Each woman in this research was assigned a pseudonym in order to maintain her anonymity. The initial interviews began with open-ended questions to elicit participants' perspectives and were designed to draw out the underlying process of decision making, which also captured the timing of decisions [34]. For example, we asked the women to describe their perspectives of: the factors (medical, physical, psychological, and family context) that influenced when decisions about risk-reducing surgeries were considered; how their relationships with family and health care professionals influenced when decisions about risk-reducing surgeries were considered; how organizations, institutions, or the health care system influenced when decisions about risk-reducing surgeries were considered; and how the appropriate time to consider risk-reducing surgeries changed over time. As data analysis proceeded, the questions became more specific to fill in gaps, explore important areas in greater depth, and verify emerging findings [34].

### Data Analysis Procedures

Data analysis involved the constant comparison of data from different participants, and across incidents and themes [34,35]. The transcripts were read numerous times and important ideas, interpretations, and themes relevant to the research questions were identified using an inductive approach [37]. Each woman's decision-making experience was captured in a narrative summary noting what risk management decisions were made and when, as well as whether these decisions changed over time. In addition, interview data related to the timing of decisions was identified, and coded for retrieval and in-depth analysis using the data management software program NVivo. One author (A.F.H.) reviewed these data to identify themes that recurred within the interviews and were evident in multiple women's accounts. During the next phase of analysis, all four members of the research team reviewed and discussed the themes. The team compared the data related to each theme, raised questions to further the analysis, and identified the relationships within and between the themes, thereby helping to refine these thematic categories. This continued until all ideas, interpretations, and themes were accounted for in the final description.

## Results

Decisions about risk-reducing surgery evolved over months and even years for many of the women in this study. At the time of being interviewed, all of the women were having biannual cancer screening through the hereditary cancer program high-risk clinic. Thirteen of 19 women with ovaries (3 women previously had bilaterally salpingo-oophorectomy to treat medical conditions) had undergone RRO and three of the 22 women with breasts had undergone RRM. The time that lapsed between receipt of genetic test results and receipt of risk-reducing surgery ranged from three months to nine years. Of the women who had not undergone risk-reducing surgery, the majority had not ruled out considering these decisions in the future. It was important to the women for decisions about RRM and RRO to be made one at a time. Moreover, the women constructed the 'right time' to consider decisions about risk-reducing surgery to be when: (1) decisions fit into their lives, (2) they had enough time to think about decisions, (3) they were ready emotionally to deal with the decisions and the consequences, (4) all of the issues and conflicts were sorted out, (5) there were better options available, and (6) the health care system was ready for them.

### One Decision at a Time

The women in this study, with one exception, did not engage in decisions about RRM and RRO concurrently. Rather, they prioritized their decisions and then considered them one at a time. The decisions the women deemed most relevant to them, less disruptive to their lives, uncomplicated, associated with fewer negative physical and emotional consequences, and supported by others, were made first. Karen, a 53-year-old woman who received her genetic test results three years previously recalled her reaction to the options of RRM and RRO:

When I saw Dr. [name] she started talking about mastectomies. And I said, "Look, I'm going to deal with the ovarian thing first, that's what has manifested in my family most and been the most lethal. And the breast thing, there's good screening tools out there and I'm not going to deal with that right this minute. I want to deal with the ovarian thing first. First things first."

In keeping with this, most of the women considered decisions about RRO first, and then RRM. The women also commonly made "temporary" decisions and then revisited their decisions later on, when they thought it was the 'right time.' Leslie, a 55 year old woman who received her genetic test results two years previously, described her approach to considering risk-reducing surgery:

Well I basically had gone from, in the summer, saying I'm going to focus on my ovaries, get that done, worry about the breast thing, and then getting really enthusiastic about maybe doing the breast stuff, going and seeing the plastic surgeon and being totally turned off, and going totally against it [RRM]. And then I guess this year it came up again, when I had another mammogram in early February and then they wanted to do a bigger magnification of it. And then that started me thinking again about this. Am I making the right decision? Is this really safe to do this route, the high surveillance? But, I've reexamined it again, and I still feel that it's premature to make a decision.

The women's experiences with cancer screening and making or following through with risk-reducing surgery decisions had a significant influence on when subsequent decisions were considered. A number of women felt "protected" from cancer because they had faith in the efficacy of screening and believed that if detected early, cancer was easily treated. The reassurance associated with screening resulted in many women feeling comfortable "sticking with screening" for the time being and postponing RRM and RRO decisions. However, anxiety, physical discomfort and experiences of finding "something suspicious" during screening prompted a revisiting of their risk-reducing surgery decisions. This was also the case when women's negative experiences with RRO resulted in their re-evaluating the 'right time' to consider RRM.

### When Decisions Fit into my Life

The women wanted to fit decisions about risk-reducing surgery into their current and future life plans. For example, Rose, a 39-year-old woman who had known her genetic test results for five years, questioned how RRM would fit into her active lifestyle:

I'm still thinking about the mastectomy [RRM]. I know that no implants can make life a little rough too. If you're an active person like me and you spend a lot of time at the pool but you have a problem with your self-image then you might not go to the pool, right? Or you might not do the sports that you might normally have done, right? And at this point I'm not spending a lot of time at the pool but pre kids I was doing triathlons and stuff. So if I get back into that kind of a lifestyle, would I be totally comfortable? So I don't think I'm ready to give that up yet.

For some, this meant postponing decisions until important events occurred, or phases of their lives completed. Younger, single women considered the importance of their ovaries to appearing youthful and breasts to appearing attractive to potential partners. The women interested in dating, starting intimate relationships, and finding a partner preferred to postpone decisions about risk-reducing surgery until they were in a serious relationship. Scarlet, a 37-year-old woman who received her genetic test results nine years ago described why RRM did not currently fit into her life:

My mom is very proactive and encourages me to go and have prophylactic mastectomy. But she's not looking at it the way I'm looking at it. It's always in the back of my mind that one day when I'm older I may do that [RRM], but not now. Not when I'm just in a new relationship and I don't even know if it will work out. It's just not the time right now.

Although most married women acknowledged that their husbands loved them for more than their breasts and ovaries, some women questioned the effects RRM and RRO would have on their relationship. It was important for these women to obtain reassurance from their partners before they seriously considered risk-reducing surgery. For some of the women who still wanted children, or who were not done childbearing, both RRM and RRO were considered premature. Christine, a 42-year-old single woman who had known her genetic test results for five years, recalled how the decision not to have children was crucial to the timing of her decision about RRO:

What I had to come to terms with mostly before I could think about the surgery [RRO] was that I wasn't going to have kids. Once I had come to terms with that, and then basically getting to the point where I don't want to have children, made it a lot easier for me. I never had a partner for very long and I don't have a lot of money. Unless you can really provide for a child it doesn't seem right.

These women felt reassured postponing their decisions about RRO because this was congruent with the hereditary cancer program recommendations to wait to make this decision until childbearing was complete. However, some women contemplated altering their life plans by looking into alternatives for having children (i.e., adoption or "freezing their eggs" and undergoing in-vitro fertilization) and engaging in family planning discussions with their partners to determine whether changing their pre-existing plans was an option.

The women also constructed the 'right time' to consider decisions about risk-reducing surgery to be when these decisions aligned with their perception of who they were at particular times and phases of their lives. This included how the women saw themselves (e.g., as younger and older), their bodies (e.g., as fertile and post-menopausal), and themselves in relation to others (e.g., as single, an intimate partner, and a widow), in the present and the future.

### When I Have Taken Enough Time to Think About Decisions

The women needed to have enough time to deliberate about risk-reducing surgery decisions so that they felt ready and able to make these decisions. Taking time to carefully consider these "big" and "complicated" decisions was not only considered by the women to be necessary, but was encouraged by health care professionals, family, and friends. Maureen, a 48-year-old woman who had known her genetic test results for five years, recalled the advice given to her by her family physician:

She [physician] just said. "You know it [RRM] is irreversible, so once you've made that decision, there's no turning back. So take your time, and weigh the pros and cons." And there are just so many pros and cons and it's huge. I mean this decision is really huge.

The women had many questions and things to think about, such as the intricate details of surgery and reconstruction, possible surgical side effects and complications, and the consequences of surgery on their feelings of femininity, body image, sexuality, and self-esteem. The 'right time' to consider these decisions was when the women had obtained answers to all of their questions, and thoroughly considered all facets of their possible options. This involved the use of a variety of decision-making approaches over time, including engaging with others, looking inwards, making sense of the numbers about risks, and weighing the pros and cons, as described in detail elsewhere [32].

The 'right time' to consider decisions about risk-reducing surgery was also when the women could concentrate their energies on making these decisions. Because some women's lives were busy, they postponed their decisions until they could give them their full attention. Dealing with other existing health problems, such as cardiovascular disease, chronic fatigue syndrome, or fibromyalgia, took precedence over considerations about preventive surgery, as did providing care to ill family members. For example, Coral, a 51-year-old breast cancer survivor, had postponed making a decision about RRM for six years because she was too busy providing care to her ailing parents and then organizing the family affairs once they passed away. "I wasn't ready [to decide about RRM] with all the responsibilities that I had to take care of. So now I just have myself and I don't have anybody to worry about."

### When I am Ready Emotionally to Deal with Decisions

The women perceived that they needed time to deal with the emotional consequences of carrying a *BRCA1/2 *mutation before they could make decisions about risk-reducing surgery. At times, the women were preoccupied with related anxiety, distress, and worry, which not only interfered with their everyday lives, but also prevented them from feeling capable of fully engaging in decision making. Christine, a 42-year-old single woman who had known her genetic test results for five years, recalled feeling too overwhelmed to engage in a yearly conversation about RRO with her physician at the hereditary cancer high-risk clinic:

It's so unconscious or irrational the kind of fear that you feel, that I didn't even show up for my appointment [at the high-risk clinic]. And that's so not like me and I was in trouble with Dr. [name]. She had to rebook and see me on a prostate day, which was not good. So that was one thing where I just kind of shut down and I think that day I just stayed home and cried and I was really upset. But nothing feels very conscious. It's very emotional.

Feeling emotionally overwhelmed led some women to feel uncertain and apprehensive about their abilities to make risk management decisions. Postponing these decisions gave the women the time they needed to cope with their emotions, get accustomed to the ideas of RRO, RRM, and breast reconstruction, and to come to terms with the consequences of these decisions. Two women sought professional psychological support to help them cope with their anxiety and depression brought on following genetic testing. The women constructed the 'right time' to consider these decisions to be when they had sufficiently addressed their anxiety, distress, and worry to be able to engage in decision making without feeling overwhelmed.

### When all of the Issues and Conflicts are Sorted Out

The women indicated that they needed to address the issues and conflicts that the decisions raised before they could finalize decisions about risk-reducing surgery. Sorting out the issues and conflicts within themselves, with their family, and with health care professionals often took time. Inner conflict was the result of the women feeling uncertain about their HBOC risk, having numerous questions about risk-reducing surgery and breast reconstruction, and encountering difficulty with weighing the pros and cons of these decisions. For example, when Lauren, a 40-year-old woman, consulted a surgeon intending to have RRO, she was informed she would have to take hormone replacement therapy post-operatively - "So then the hormones would increase my chance for breast cancer, and so there was all that conflict." Although gathering information and advice was helpful, at times this resulted in obtaining new information that needed to be considered in decisions and added further complexity to decision making. In these situations, women were often left feeling more confused and conflicted about the decisions they were trying to make. For example, Leslie, a 55 year old woman who received her genetic test results two years previously, described feeling conflicted about breast reconstruction upon learning about the details of the procedure:

But then I find out that they put it [the implant] behind your pectoralis major muscle. They don't put it in your skin where your breast was. They put it behind your pectoralis major, between that and your chest wall. So then they have to fill this thing with water and stretch the muscle every two weeks. That's what the expansion thing is. They say it's for your skin on all the websites. No, it's for the muscle, to accommodate this lump. And I just thought that's ridiculous. I couldn't believe that's what they did. So now I don't know what to do.

There were also issues and conflicts involving family members that the women wanted resolved before they felt comfortable finalizing their decisions. Some family members voiced their concerns about risk-reducing surgery, while others expressed their opinions about the right course of action for the women to take. Wanting to remain sensitive to family members concerns and opinions, some women postponed their decisions or followed through with their decisions at a later time. For example, Lilith, a 39-year-old woman, decided to have RRO and RRM a couple of months after she was found to carry a *BRCA1/2 *mutation. But, she postponed RRM indefinitely and RRO for over a year because her mother was upset and disagreed with her decision. Sorting through family conflict also involved taking the time to provide explanations and information, and to try and convince family members that the decision the women were leaning towards was the best decision for them.

Sorting through issues with health care professionals was also an essential step to settling on decisions about risk-reducing surgery. Seeking second and third medical opinions in order to investigate alternative options, double check recommendations, gather more information, and obtain advice took time. Furthermore, these interactions complicated decisions when these professionals provided advice that conflicted with the recommendations or advice they previously received. Often times, disagreements and conflict with health care professionals stemmed from disparate views about what was the most important, acceptable, or desirable decision. For example, at the time of interview Coral's decision to have RRM and reconstruction had been prolonged approximately six months because her preferences for reconstruction differed from those of her physician:

Right now we're arguing about the size [that my reconstructed breast should be]. He [physician] says well, you know with the breadth of your chest you have to be a C cup. And I'm going, "Oh please, make them smaller because you have no idea what it's like. You carry twenty pounds on your chest and then tell me that you want me to be the same size." Well I have the surgeon on my side now. She says, "It's men, you know what it's like." I mean he is the professional so I said, "Why do you think I'll look freakish? I want a B cup. I see no difference between a B or a C cup as far as my chest wall goes." So I'm just goanna stick with it.

### When there are Better Options

Some women thought the time was not yet right to consider making decisions about risk-reducing surgery because not enough was known about hereditary cancer. This was reflected in the comments made by Jen, a 53-year-old woman who had known she carried a *BRCA1/2 *mutation for five years: "I mean we're still talking about having a mastectomy in the future, but I don't have any breast cancer in my family so I'm thinking maybe they don't know enough about these genetic mutations yet." The women considered the cancer genetics field to be a young science characterized by new technology, numerous unanswered questions, and evolving knowledge. Reflecting on the past, the women perceived there to be more options now than ever before to reduce their risk of developing or dying from HBOC, and they expected this trend to continue. As a result, the women had faith in scientific and medical progress and perceived the 'right time' to consider decisions about risk-reducing surgery to be when new scientific evidence emerges and less invasive, less drastic, better tolerated, and more acceptable medical and surgical options and techniques become available and accessible. For example, Leslie described her decision to wait to consider RRM until different breast reconstruction techniques were offered in Canada:

I've got another friend who has the gene. She's from Iran and her sister in Iran had breast cancer, and had the mastectomy and reconstructive surgery. And over there they have a very highly sophisticated plastic surgery industry, which I didn't know about, and she just had the implant put underneath the skin, and it was fine. So I'm just on hold until they start doing that [reconstructive surgery] here.

Some women also relied on the guidance of experts to help them determine when better options would warrant revisiting their previous decisions. This was evident in the explanation provided by Maurice, a 65-year-old woman who received her genetic test results nine years previously:

The consensus of the cancer program and my doctor was that it [RRM] wasn't necessary. And if it's not necessary I'm not going to run out and do it. But if anyone thought it would really be a good idea for me, I'm prepared to do it. I have made the decision to stick with screening for now, and I'm letting that decision sit until I hear from someone who knows that there could be a better decision.

### When the System is Ready for Me

The women's constructions of the 'right time' to consider decisions about risk-reducing surgery were also influenced by perceptions about when the Canadian health care system could accommodate their choice. A number of women engaged in ongoing conversations about risk-reducing surgery during biannual breast cancer screening appointments at the hereditary cancer high-risk clinic. They had faith in the existing referral processes to access further services that would help them decide about RRM and RRO. Most women were not upset that referrals to surgeons took up to a year because this gave them time to think more carefully about risk-reducing surgery, as evident in Coral's description:

When I saw Dr. X at the cancer agency for my check up she said, "Well, I can set you up to talk to the surgeon and read some information on it." And if I wanted to she could put me in touch with other people who had the surgery. A lot of time went by in-between but I wasn't ready. Waiting to have the chat, I pretty much had time to think about it. And I knew that was what I was going to do. I think my mind was made up before I even saw the surgeon.

Wanting to "get things over and done with" once they had made their decisions, some women felt disappointed and anxious when the system was not ready for them. The lack of seamless care also led some women to postponed their decisions, believing the Canadian health care system could not meet their needs, as described by Lauren, who knew her *BRCA1/2 *carrier status for four years:

My decision process, and my sister's decision process, would be a lot different if we could have the reconstruction done at the time as the mastectomy. It's huge. My sister actually has opted to have both of her breasts removed prophylactically and to have the reconstruction later, and she's been on a waiting list for two and a half, three years now. It just doesn't make sense to me to do the surgeries twice. And why can't we go with dignity and grace? You know, if I'm ever able to have reconstruction at the same time, I'd seriously consider the surgery. It would make my decision easier.

## Discussion

The study findings described how women who carry a *BRCA1/2 *mutation construct the 'right time' to make decisions about RRM and RRO. It was important for the women in this study to consider decisions in the context of their lives, take enough time to deliberate, cope with their emotions, sort through issues and conflicts, wait for better medical and surgical options, and factor in the readiness of the health care system. These findings provide possible explanations for the time frames between genetic testing and risk-reducing surgeries observed in other studies [6-9].

Of note, the women in this study did not frame their decision making as "delayed," "slow," or taking "too long". This differs from researchers and clinicians who are of the opinion that risk-reducing surgery is the best way to eradicate breast cancer risk in *BRCA1/2 *mutation carriers and have voiced their concern that some women are waiting too long to decide about RRM and RRO [38]. These conflicting perspectives appear to stem from different concerns and priorities associated with HBOC risk management. On the one hand, some women perceive prolonging or deferring decisions about risk-reducing surgery as enhancing their quality of life and helping them manage the inherent decisional conflict. On the other hand, there are also risks with delaying these decisions that cannot be ignored. These women could develop cancer in the interim. Future research is needed to understand whether these different concerns and priorities complicate the provision of genetic services, and if so, in what capacity.

McCullum and colleagues [31] reported that women prolonged their RRM decisions while they focused on quality of life issues, specifically the associated risk of decreased physical and emotional well-being. For the women in the present study, the physical consequences associated with risk-reducing surgery held different meanings at different phases of their lives. Consistent with the genetic testing and risk management literature [16,17,39-41], younger women considered decisions about RRO and RRM inappropriate because of their life plans, such as finding a life partner, childbearing and childrearing. Yet some of the women contemplated changing these life plans or searching out alternatives, phenomena previously reported but requiring further examination [16]. It is possible that these women were attempting to reconcile their competing desires to have risk-reducing surgery sooner than later, but also fulfill important life goals essential to their quality of life. Attending to dimensions of self other than physical health has also been found to an important part of the decision-making process [32].

Emotional well-being also influenced when the study participants considered risk-reducing surgery decisions. These women needed time to deal with the emotional consequences of carrying a *BRCA1/2 *mutation and consider the implications of risk-reducing surgery before making final decisions. Rather than rush into decisions to cope with their distress, anxiety, and worry, these women prolonged the decision-making process while they coped with their emotions and also postponed their decisions until they felt emotionally ready. Women have previously reported declining genetic testing because of their desire to protect themselves and their family from the emotional consequences [42]. In contrast, research that examined predictors of RRM and RRO found anxiety, distress, and worry to be key predictors of risk-reducing surgery [24-30]. Women have described their breasts and ovaries as "time bombs" and risk-reducing surgery represented a strategy for managing the associated worry and anxiety [15,43]. These disparate findings suggest that emotions of anxiety, distress, and worry do not always predispose women to make particular decisions at particular times following genetic testing. Instead, emotions play a much more complex role in the timing of women's decisions related to RRM and RRO.

In the study by Kenen and colleagues [44], women who carried a *BRCA1/2 *mutation vacillated from one position to the other and took years to make up their minds about RRM. Most of these women reported spending a great deal of thought on RRM during this decision-making process. The research by Kenen et al. [44], in combination with findings from the present study, suggests that decisions about risk-reducing surgery are fraught with decisional conflict. Decisional conflict occurs when there are: risks or scientific uncertainty about the benefits and harms, choices with large potential gains and losses, value tradeoffs in selecting a particular course of action, and potential regrets with the selected option [45]. Tan and colleagues [46] found that the two most important reasons for women to postpone RRM were uncertainty about proceeding with surgery and the need for more risk information. The lack of conclusive information about the risk of HBOC and the implications of risk-reducing surgery were particularly problematic for the women in the present study, as reported in other research [28,31,43,47]. It is, therefore, unsurprising that these women struggled with uncertainty and were compelled to seek out second and third medical opinions over time. Further research is needed to understand the impact of these opinions and advice on women's uncertainty and the decision-making process, as well as how women resolve conflict when differing opinions are garnered.

The research that has focused on decisions about *BRCA1/2 *genetic testing suggests that family members are intimately involved in the genetic testing process, before, during, and after their relative decides to go forward with testing [48]. Family relationships are also affected when women are found to carry *BRCA1/2 *mutations [48,49]. Researchers have reported that women who tried to discuss hereditary cancer or risk-reducing surgery with their families have been met with resistance, shock, hostility, and additional negative responses [40,50,51]. This resulted in women self-censoring by pulling back or not talking about cancer for fear of causing worry or anxiety [51]. In a similar fashion, the women in this research further postponed making decisions about risk-reducing surgery because they wanted to remain sensitive to family members' concerns and opinions. They also attempted to resolve family conflicts and disagreements before they felt comfortable making RRM and RRO decisions. Future research exploring family dynamics in relation to decisions about risk-reducing surgery would broaden our understanding of these findings.

International differences in the uptake rates of risk-reducing surgeries have been observed and attributed to health care providers' recommendations and continuity of follow-up, as well as cultural variations [52]. For example, in one study, 344 women who attended a cancer genetics clinic in Canada (Quebec), Britian or Great Britian were found to vary in their preferences regarding cancer prevention [53]. The authors attributed this variation to cultural differences between countries. Although it was unclear in this study whether culture was an influential factor, it was apparent that the structure of Canadian health services plays a significant role in when women consider risk-reducing surgery, as do the barriers women encounter accessing specific services that they believe would best meet their needs. Although timely and accessible health care for all Canadians is a cornerstone of publicly funded health care, patients routinely encounter significant challenges accessing and receiving timely, coordinated, and comprehensive care from interprofessional teams [54]. It appears hereditary cancer and preventive surgical services are no exception. In the present study, the extended decision-making process was in part a by-product of the health care system in Canada, wherein the women encountered lengthy wait times for referrals as well as surgery and breast reconstruction. The finding that this time lag gave some women needed time to think more closely about risk-reducing surgery suggests that women are still engaged in the decision-making process and possibly preparing for consultations while they wait. Further inquiry into the feasibility of offering decision-making support during this time is needed.

There are limitations inherent to this research. This study included participants who were recruited from a single hereditary cancer centre in Canada. Caution must therefore be exercised when determining the relevance of the findings in other settings. As well, the perspectives of older women are underrepresented in this study since only one woman was over the age of 65. Moreover, we did not access any participants with positive *BRCA1/2 *test results who chose not to partake in the recommended HBOC screening. It is possible that the study participants represent a homogeneous group with ready access to screening services and beliefs in early detection. There might also be women who are considering decisions about RRM and RRO who do not have contact with health care professionals. Genetic information, evidence about the effectiveness of HBOC risk management strategies, and the availability of online and written resources about risk-reducing surgery has changed over the years. Thus, women who received their *BRCA1/2 *test results and were availed of the options of RRM and RRO at different times, particularly a number of years ago, might have had different kinds of experiences than women who received their genetic test results more recently.

## Conclusion

In conclusion, women's perspectives about the appropriate time to consider decisions about risk-reducing surgeries have been absent from the scientific literature to date. Incorporating women's perspectives on the timing of these decisions could provide valuable direction for decision support. The above findings provide evidence that decision support ought to be accessible when women want and need the support because some women prolong or defer decisions to a later time. For those who are overwhelmed with simultaneously facing multiple decisions about HBOC risk management, it may be beneficial to help women prioritize their needs and to work through each of their decisions when they feel ready. Some women may require ongoing psychological support because they find it difficult to come to terms with their HBOC susceptibility, as well the ideas of RRM and RRO. Making this option available to those women who are interested is crucial. The findings suggest that women consider decisions about risk-reducing surgery in the context of their life plans, relationships, medical science, and the health care system. This provides evidence that broader approaches to decision support, such as relational or women-centered approaches, might complement existing services. A relational approach could assist women with mobilizing support and resources, communicating with others about HBOC risk management, and working through interpersonal issues. A women-centred approach addresses issues beyond traditional medical interventions, placing health in its broad social context, and also addresses barriers to access and respects women's diversity [55]. Although risk-reducing surgery decisions are women's decisions, women should not be saddled with the burden of tackling barriers to accessing health care services. Health care professionals, health care organizations, and government must work hard to resolve these challenges.

## Competing interests

The authors declare that they have no competing interests.

## Authors' contributions

AFH carried out this research as part of her doctoral dissertation. She designed the study, collected and analyzed the data, and drafted the manuscript. JLB and LGB participated in the formulating and conducting of the research, as well as the presentation of ideas in this manuscript. CKS provided consultation with the design of the research, and reviewed and provided significant feedback on this manuscript. All authors read and approved the final manuscript.

## Pre-publication history

The pre-publication history for this paper can be accessed here:

http://www.biomedcentral.com/1472-6874/10/24/prepub
